# PINK1 regulates apoptosis of osteosarcoma as the target gene of cisplatin

**DOI:** 10.1186/s13018-023-03615-w

**Published:** 2023-02-23

**Authors:** Zhenxing Si, Zilong Shen, Feiyu Luan, Jinglong Yan

**Affiliations:** 1 Department of Emergency Surgery, The First Affiliated Hospital of Harbin Medical University, Harbin, China; 2 Department of Orthopedic Department, The Second Affiliated Hospital of Harbin Medical University, 246 Xuefu Road, Nangang District, Harbin, 150001 Heilongjiang China

**Keywords:** Osteosarcoma, PINK1, FOXO3a, Apoptosis, Cisplatin

## Abstract

**Background:**

Osteosarcoma is a common primary bone malignancy prevalent among adolescents and young adults. PTEN-induced kinase 1 (PINK1) regulates Parkinson's disease, but its role in cancers is unknown.

**Objective:**

This study was designed to analyze the mechanism by which PINK1 affects osteosarcoma using bioinformatics and cell experiments.

**Materials and methods:**

The gene expression profiles were downloaded from the TARGET database. Several online databases were used to analyze the expression and protein‒protein interaction networks. CCK-8 cell viability assays and cisplatin treatment were used to assess cell activity with or without cisplatin treatment. Acridine orange/ethidium bromide (AO/EB) fluorescence staining was used to calculate the percentage of apoptotic cells.

**Results:**

Through bioinformatics analysis, we found that high expression of PINK1 was associated with poor prognosis in patients with osteosarcoma, and PINK1 inhibited apoptosis and promoted proliferation pathways. Next, we found that both PINK1 mRNA and protein levels were upregulated in osteosarcoma tissues. Additionally, we found that PTEN was reduced, while FOXO3a was markedly increased in osteosarcoma, suggesting that FOXO3a and not PTEN induced the overexpression of PINK1. CCK-8 and clonogenic assays showed that the knockdown of PINK1 decreased the growth of U2OS osteosarcoma cells. Ki67 immunofluorescence staining revealed that reduced cell proliferation in U2OS cells resulted in the depletion of PINK1. In addition, our AO/EB staining results indicated that the knockdown of PINK1 resulted in an increase in apoptotic cells and increased the levels of cleaved caspase-3. Furthermore, our experiments revealed that cisplatin promotes OS cell apoptosis by downregulating PINK1.

**Conclusion:**

Collectively, our findings demonstrate that PINK1 is crucially involved in osteosarcoma and suggests that it can promote the apoptosis of OS cells as the downstream target gene of cisplatin.

## Introduction

Osteosarcoma constitutes the most frequent childhood primary bone tumour that most frequently arises in the metaphyseal ends of long bones [1]. In recent decades, the survival rate of osteosarcoma patients has remained low [2]. Numerous genes that may play vital roles in osteosarcoma development, as well as progression, have been investigated [3]. However, there is an urgent need to explore new therapeutic targets and approaches to improve survival outcomes. PTEN-induced kinase 1 (PINK1), a serine/threonine kinase, was originally discovered in cancer cells [4]. Mutations in PINK1 that lead to loss of function cause autosomal recessive Parkinson’s disease, and there is sufficient evidence of the role of PINK1 in neuronal and other cell systems [5]. Typically, PINK1 is ubiquitously expressed and is a significant modulator of mitochondrial quality control, encompassing fusion, bioenergetics, fission, and mitophagy [6]. Research evidence has shown that both PTEN and FOXO3a induce PINK1 expression. Additionally, PINK1 has cytoprotective as well as anti-apoptotic functions [7]. PINK1 is a critical regulator of mitophagy that recruits autophagy receptors, such as NDP52 and optineurin, to induce mitophagy. Recently, PINK1 has gained increased attention as an essential modulator of the cell cycle in cancers, including glioblastoma, melanoma, and breast cancer, among others, suggesting its prospective role in tumorigenesis [8]. However, the biological function and related mechanisms of PINK1 in osteosarcoma remain unknown.

In this study, we found that PINK1 was upregulated in osteosarcoma and that FOXO3a rather than PTEN modulated its expression. The knockdown of PINK1 reduced cell proliferation, and PINK1 silencing induced apoptosis in U2OS osteosarcoma cells. Additionally, we observed that cisplatin promotes OS cell apoptosis by downregulating PINK1. Altogether, we demonstrate that PINK1 plays a critical role in osteosarcoma and constitutes a potential therapeutic target for osteosarcoma.

## Materials and methods

### GEPIA database

GEPIA2 database (http://gepia2.cancer-pku.cn/#general, [9]) is a newly developed interactive web server for analyzing the RNA sequencing expression data of 9736 tumors and 8587 normal samples from the TCGA and the GTEx projects, using a standard processing pipeline. The GEPIA2 database combines data from the TCGA and GTEx databases to enable users to explore the relationship between gene expression and clinical manifestations. In this study, we explored the differential expression of PINK1 across cancers and normal tissues using the GEPIA2 database, ANOVA method was used for comparison with the following threshold values: |log2FC| cutoff = 1, Log Scale = log2 (TPM + 1) and q-value cutoff = 0.01[10].

### Survival analysis

Osteosarcoma patient data from the TARGET database (https://ocg.cancer.gov/programs/target) were divided into PINK1-high and PINK1-low based on the median gene level of PINK1. The survival rates of the two groups were compared using Kaplan‒Meier survival curves and log-rank analysis. The R packages “ggpubr” and “limma” were used for clinicopathological correlation analyses.

### Protein‒protein interaction network

STRING database (https://www.string-db.org/, [11]) is a database of known and predicted protein–protein interactions. The interactions include direct (physical) and indirect (functional) associations; they stem from computational prediction, from knowledge transfer between organisms, and from interactions aggregated from other (primary) databases. We investigated the proteins that interact with PINK1 using the STRING database. The species was set to human with a confidence greater than 0.4 and no more than 20 interacting proteins.

### Gene set enrichment analysis

Based on PINK1 median expression, osteosarcoma patient data were categorized into high- and low-expression groups. GSEA is a method based on functional categories that could calculate the enrichment score of gene sets and discover different functional phenotypes. We used GSEA to compare the biological pathways between the two groups. We performed enrichment analysis using GSEA software (4.1.0) from the Molecular Signatures of database (http://www.gsea-msigdb.org/gsea/msigdb/index.jsp). |NES|> 1, *p* < 0.05, FDR < 0.25 were considered statistically significant.

### Clinical tissues

We collected the osteosarcoma tissues as well as the corresponding adjacent tissues from patients at the Third Affiliated Hospital of Harbin Medical University. The subjects provided informed consent, and the Ethics Committee of Harbin Medical University approved the research method. After collecting the tissue samples, we immediately froze them in liquid nitrogen for preservation until use.

### Cell culture

We cultured osteosarcoma U2OS cells in DMEM containing 10% foetal bovine serum, 50 U/ml penicillin, and 50 µg/ml streptomycin (Invitrogen, USA). We kept all the cells in a humidified incubator (37 °C, 5% CO2).

### Plasmid and siRNA transfection

We seeded U2OS cells into 35 mm plates 24 h prior to transfection. For PINK1 overexpression, we used Lipofectamine 2000 (Invitrogen, USA) to transfect 4 μg of pcDNA3.1-PINK1 vector or empty vector into U2OS cells. For PINK1 silencing, we transfected PINK1 siRNA or control siRNA using Lipofectamine 2000 (Invitrogen, USA) with serum-free medium as per the manufacturer’s instructions. Five hours after transfection, we changed the cells to complete medium and then cultured them for 48 h.

### RNA extraction and real-time PCR

We extracted total RNA according to the manufacturer's protocol using TRIzol reagent (Invitrogen, USA). Then, using Superscript II (Invitrogen, USA), we reverse-transcribed the RNA into cDNA. Quantitative PCR was performed on a 7500 Real-Time PCR System (Applied Biosystems, USA) using Power SYBR Green PCR Master Mix (Life Technologies, USA). The settings of qPCR were 30 cycles at 95 °C for 30 s, 60 °C for 30 s, and 72 °C for 35 s. We used the following primers for qPCR:

- PINK1-F: 5′-GCCTCATCGAGGAAAAACAGG-3′;
- PINK1-R: 5′-GTCTCGTGTCCAACGGGTC-3′;
- GAPDH-F: 5′-AGCCTCCCGCTTCGCTCTCT-3′;
- GAPDH-R: 5′-GCGCCCAATACGACCAAATCCGT-3′.

We used GAPDH as our housekeeping gene.

### Antibodies and western blotting

We used RIPA lysis buffer containing a cocktail of protease inhibitors (Roche, Switzerland). Next, we separated the proteins using SDS‒PAGE and transferred them to a nitrocellulose membrane (Pall Corporation, USA). Then, we probed the blots with primary antibodies against actin (1:2000), Bax (1:1000), Bad (1:1000; Santa Cruz, USA), and cleaved caspase-3 (1:1000). The membranes were then washed and incubated with secondary antibodies (1:10,000; Cell Signalling, USA) and then visualized with ECL reagent (GE Healthcare, USA).

### CCK-8 cell viability assay and cisplatin treatment

We seeded U2OS cells in 96-well plates at a density of 2 × 10^3^/well and cultured them for 72 h after treatment with PINK1 siRNA or control siRNA. Then, we evaluated viability using Cell Counting Kit-8 (Dojindo, Japan).

The CCK-8 analysis method was used to observe the effect of cisplatin on the IC50 of U2OS cells. We seeded U2OS cells into a six-well plate and allowed a growth ratio of 50–60%. Subsequently, we harvested U2OS cells treated with the IC50 concentration of cisplatin for 24 h.

### Clonogenic survival assay

We counted 8 × 10^2^ U2OS cells transfected with PINK1 siRNA or control siRNA. After 10 d of culture, the cells were stained with 0.1% crystal violet in 20% methanol for 15 min. We then photographed the samples and counted the visible colony numbers.

### Fluorescent staining of acridine orange/ethidium bromide (AO/EB)

U2OS cells were transfected with PINK1 siRNA or control siRNA for 48 h. Then, the cells were incubated with a mixture of acridine orange and ethidium bromide for 5 min (Solarbio Biotechnology, China). We observed the morphological changes in the cells with a 200 × fluorescence microscope. To obtain the percentage of apoptotic cells, we used the formula: Apoptosis rate (%) = Number of apoptotic cells/total number of cells in the count.

### Flow cytometry assay

U2OS cells transfected with PINK1 siRNA or control siRNA were fixed with 2% cold formaldehyde and stained with 5 µg/µl 7-AAD. Then, the cell cycle was measured using a BD Accuri™ C6 flow cytometer (BD Biosciences, USA). ModFit LT™ Trial and Reader Version 5.0 software were utilized to analyze the data.

### Data analysis

Each measured value was taken from 3 to 6 independent experiments and expressed as the mean ± SEM. We used a 2-tailed test for pairwise comparisons (GraphPad Prism version 5). *p* < 0.05 was considered statistically significant.

## Results

### Bioinformatic analysis of the potential function of PINK1 in osteosarcoma

**Fig. 1:**
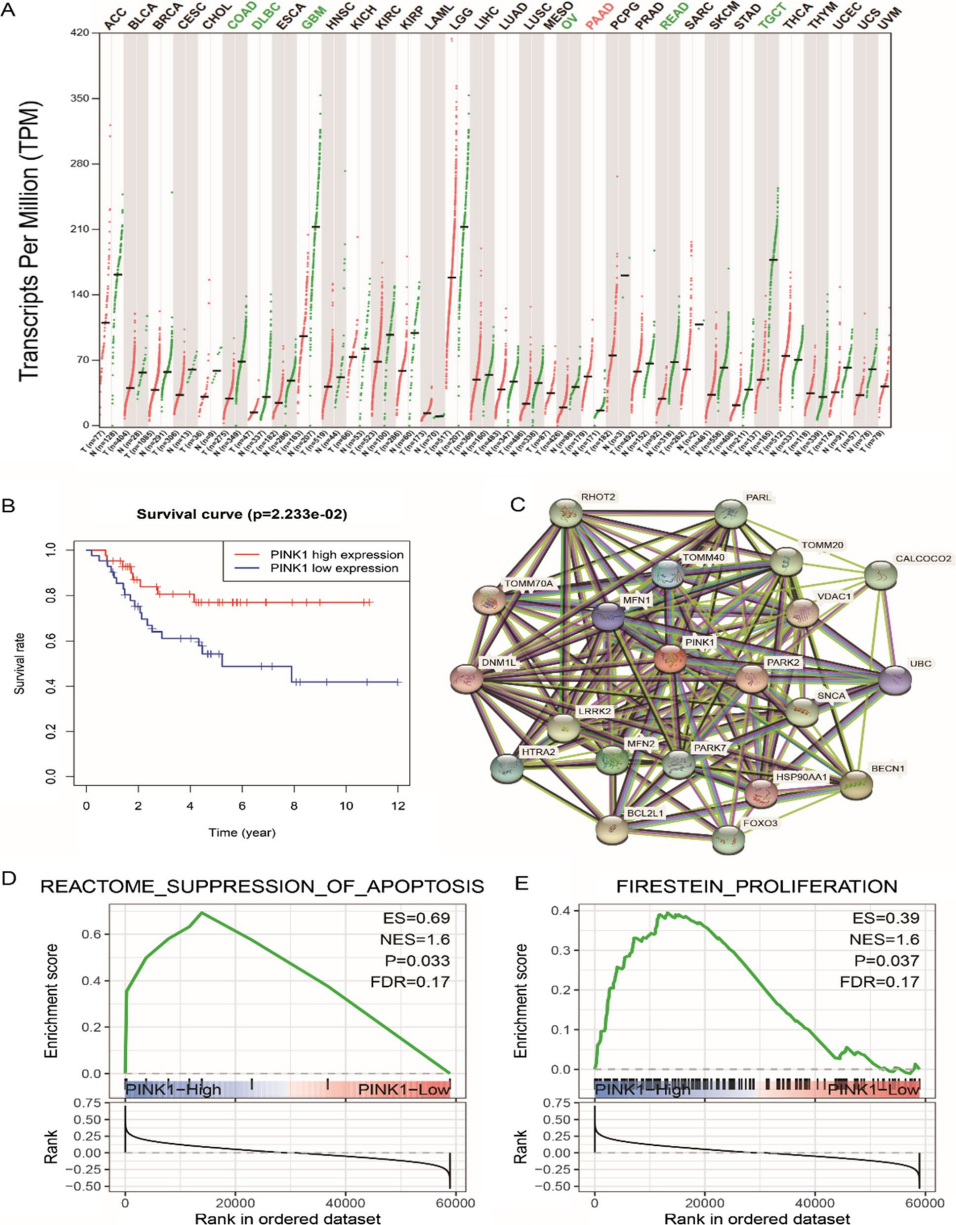
Bioinformatics analysis of PINK1. **A** Pan-cancer expression profiling analysis of PINK1-based GEPIA2 database. **B** Overall survival analysis of PINK1 in osteosarcoma patients. **C** Protein interaction network of the top 20 co-expressed genes for PINK1. **D**, **E** Gene Set Enrichment Analysis (GSEA) of PINK1. PINK1 high expression groups exhibited enrichment of REACTOME SUPPRESSION OF APOPTOSIS (**D**) and FIRESTEIN PROLIFERATION (**E**).

The role of PINK1 in cancers such as osteosarcoma is still unclear. Differential expression analysis of PINK1 in pancarcinomas showed that compared with the corresponding normal tissues, PINK1 expression was downregulated in COAD, DLBC, GBM, OV, READ and TGCT but upregulated in PAAD (Fig. 1A). However, analysis of its expression in osteosarcoma is lacking. A survival analysis performed based on data from the Target-OS dataset showed that high PINK1 expression was associated with poor prognosis (Fig. 1B). Using the STRING database, we constructed a PPI (protein‒protein interaction) network based on the expression of PINK1, and the results showed that PINK1 interacted with 20 proteins, such as FOXO3 (Fig. 1C). GSEA enrichment analysis of all curated gene sets showed that the high expression of PINK1 was positively correlated with the inhibition of the apoptotic pathway and proliferation pathway (Fig. 1D, E). In summary, the bioinformatics results showed that PINK1 expression was differentially expressed in multiple carcinomas. Its high expression was associated with poor prognosis in patients with osteosarcoma, which may be related to the inhibition of PINK1 on the apoptosis pathway and the promotion of the proliferation pathway.

### Upregulation of PINK1 in osteosarcoma is regulated by FOXO3a

**Fig. 2:**
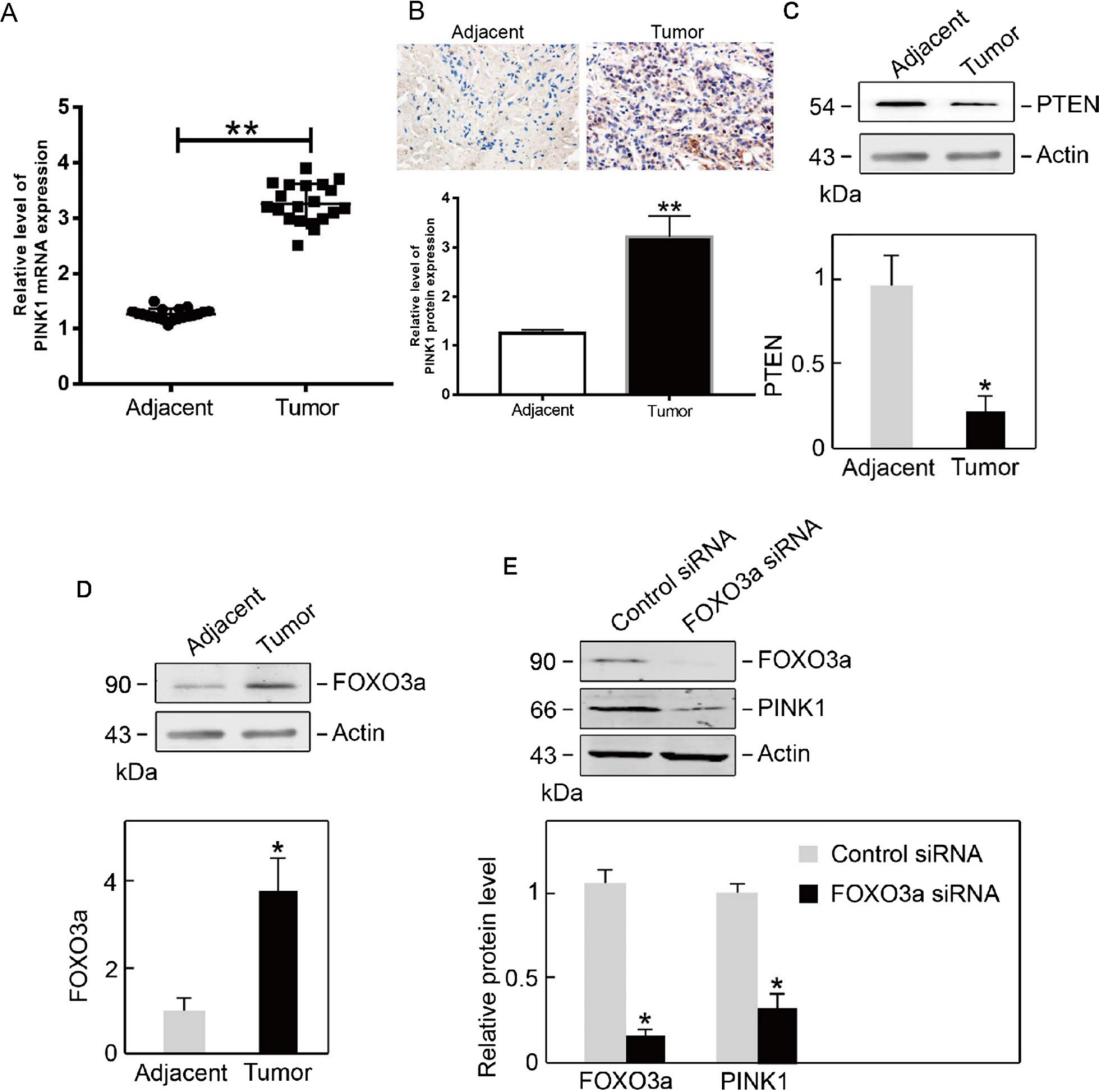
Elevated expression of PINK1 in osteosarcoma is regulated by FOXO3a in osteosarcoma. **A** PINK1 mRNA levels were elevated in osteosarcoma tissue samples. **B** PINK1 protein levels were elevated in osteosarcoma tissue samples. **C** PTEN expression was reduced in osteosarcoma tissue samples. **D** FOXO3a expression was increased in osteosarcoma tissue samples. **E** Depletion of FOXO3a suppressed PINK1 expression. *p < .05 versus control.

To establish the expression of PINK1 in osteosarcoma cells, we collected 20 pairs of osteosarcoma tissue samples and the corresponding adjacent samples. Subsequently, we used qRT‒PCR and immunohistochemistry to assay the mRNA and protein levels of PINK1, respectively. Both mRNA and protein levels were upregulated in osteosarcoma tissues but not in adjacent tissues (Fig. 2A, B). Previous studies have shown that PTEN induces PINK1 expression. Thus, we analyzed the protein expression of PTEN in osteosarcoma tissues. Interestingly, we found that the level of PTEN in osteosarcoma was significantly lower than that in adjacent tissues, which is contrary to the elevated PINK1 expression (Fig. 2C). Furthermore, we found that FOXO3a was markedly increased in osteosarcoma tissues compared with adjacent tissues (Fig. 2D), suggesting that overexpression of PINK1 might be regulated by FOXO3a. This is also consistent with the results of bioinformatic analysis, which showed a protein interaction between PINK1 and FOXO3. To test this hypothesis, we transfected FOXO3a siRNA into U2OS osteosarcoma cells. The western blot results demonstrated that FOXO3a siRNA significantly repressed FOXO3a expression (Fig. 2E). Simultaneously, the protein level of PINK1 was considerably reduced (Fig. 2E). Altogether, these results indicate that FOXO3a modulates the overexpression of PINK1 in osteosarcoma cells.

### Silencing of PINK1 suppresses osteosarcoma cell proliferation.

**Fig. 3:**
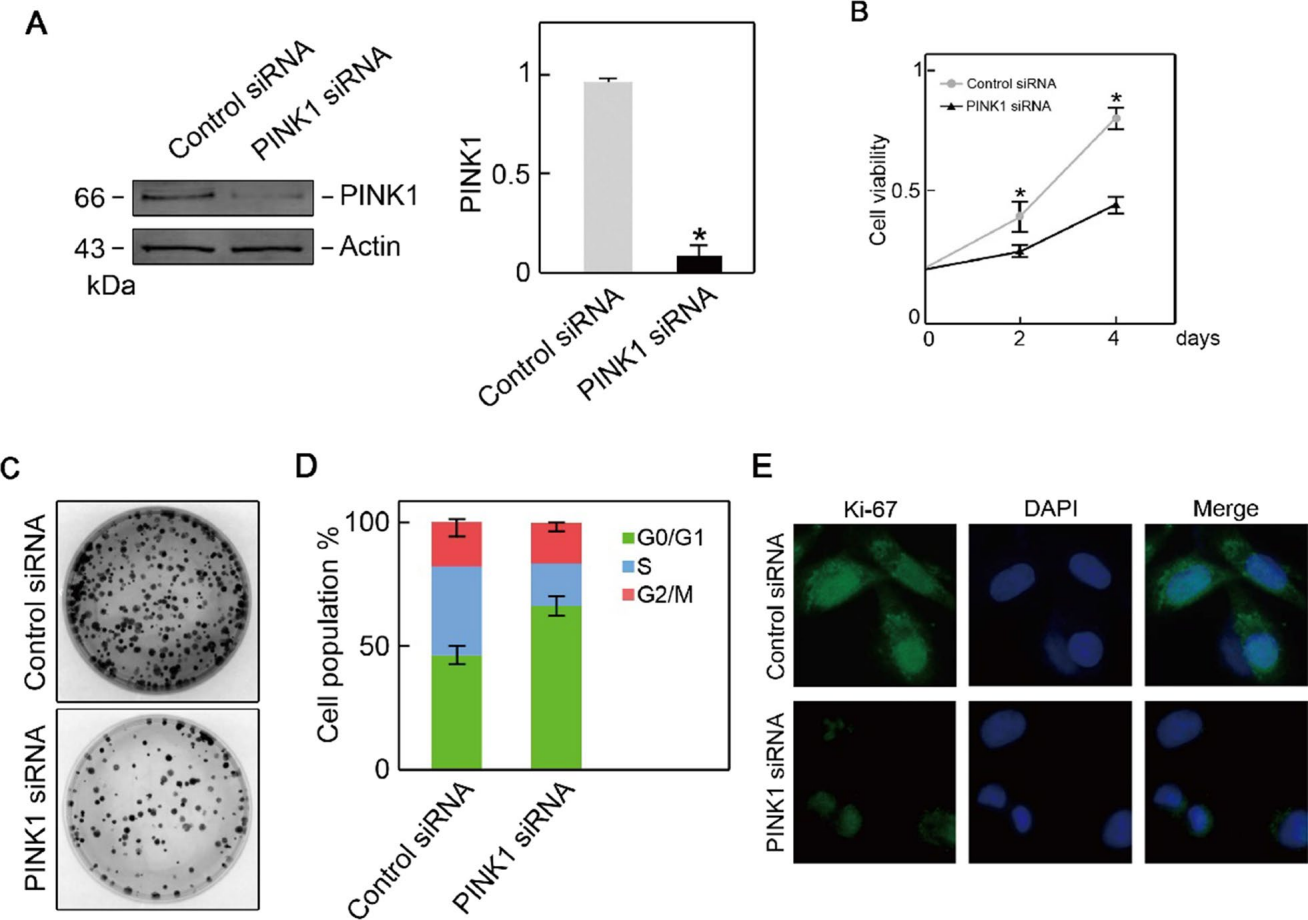
Silencing of PINK1 suppresses osteosarcoma cells proliferation. **A** PINK1 siRNA successfully inhibited PINK1 expression in U2OS cells. **B** Cell growth was suppressed by PINK1 siRNA transfection in U2OS cells. **C** Knockdown of PINK1 decreased colony formation in U2OS cells. **D** Knockdown of PINK1 arrested cell cycle at G1/S in U2OS cells. **E** Knockdown of PINK1 showed lower Ki-67 immunofluorescence staining in U2OS cells.

PINK siRNA- or control siRNA-transfected U2OS cells were used as an in vitro model to explore the biological function of PINK1 in osteosarcoma. As expected, siRNA transfection markedly suppressed PINK1 expression (Fig. 3A) as well as the growth of U2OS cells, as assessed by CCK-8 assay (Fig. 3B). The clonogenic assay results showed reduced colony numbers upon PINK1 silencing (Fig. 3C). This observation was further confirmed by a flow cytometry assay showing G1/S arrest upon PINK1 knockdown (Fig. 3D). The immunofluorescence staining of Ki-67 (proliferation marker) exhibited diminished signals in the PINK1-depleted U2OS cells compared to control siRNA-transfected U2OS cells (Fig. 3E), suggesting that PINK1 silencing decreased U2OS cell proliferation capability. Altogether, these results indicate that silencing PINK1 inhibits osteosarcoma cell proliferation.

### Silencing of PINK1 promotes apoptosis in U2OS cells

**Fig. 4:**
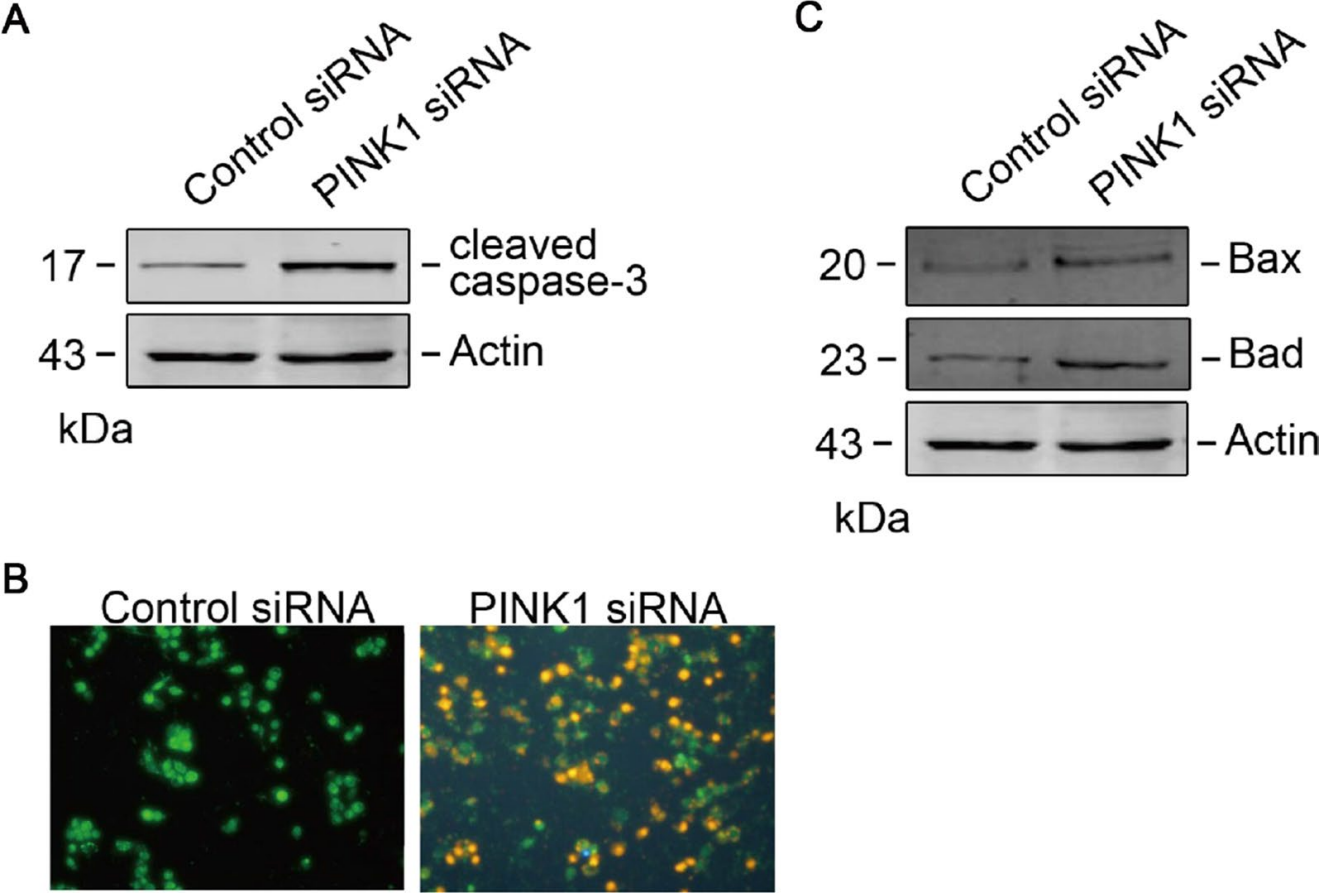
Silencing of PINK1 promotes apoptosis in U2OS cells. **A** Silencing of PINK1 induced caspase-3 cleavage in U2OS cells. **B** Silencing of PINK1 increased apoptosis in U2OS cells assessed by AO/EB staining. **C** Silencing of PINK1 increased pro-apoptotic protein expression.

Our western blot results showed remarkably elevated levels of cleaved caspase-3 following PINK1 suppression (Fig. 4A), indicating elevated apoptosis in PINK1-depleted U2OS cells. We verified these results using AO/EB staining (Fig. 4B). Additionally, proapoptotic proteins, including p53, Bad, and Bax, were upregulated by PINK1 suppression in U2OS cells (Fig. 4C). These results demonstrate that silencing PINK1 promotes apoptosis of U2OS cells.

### Cisplatin regulates U2OS apoptosis by downregulating PINK1 expression

**Fig. 5:**
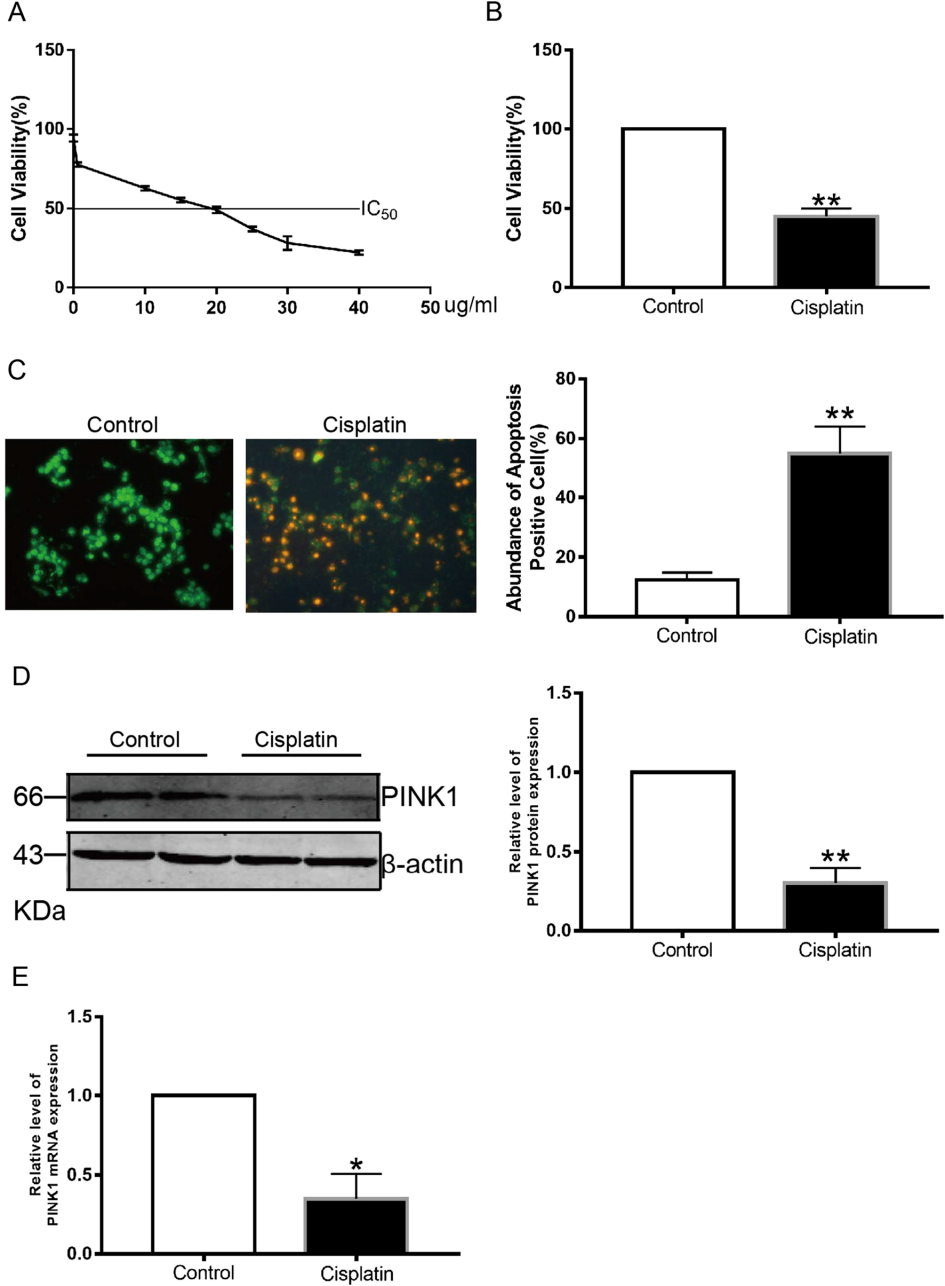
PINK1 is down-regulated in OS cells after cisplatin treatment. **A** U2OS cells with different concentrations of Cisplatin. **B**, **C** Cisplatin could significantly inhibit the U2OS cell growth and apoptosis. **D**, **E** Western blot and qRT-PCR was assessed to compare PINK1 expression in U2OS cells before and after Cisplatin treatment. *p < .05, **p < .01.

Cisplatin is a cell cycle nonspecific drug that inhibits DNA replication and has been widely used in postoperative chemotherapy for various cancers, including OS [12]. It has been reported that cisplatin can induce apoptosis of cancer cells through both endogenous and exogenous pathways, but the downstream target genes of its effects remain unclear [13]. To detect the effect of cisplatin on cell proliferation, U2OS cells were treated with different concentrations of cisplatin (0, 10, 20, 30, 40, or 50 μg/mL), and the IC50 of cisplatin was 18.70 μM (Fig. 5A). Subsequently, U2OS cells were treated with cisplatin at the IC50 concentration for 24 h, and cisplatin significantly inhibited the proliferation of osteosarcoma cells (*p* < 0.05, Fig. 5B). In addition, cisplatin at the IC50 concentration significantly promoted apoptosis of U2OS cells (*p* < 0.05, Fig. 5C). At the same time, PINK1 expression was detected in the cisplatin-treated group and was found to be significantly decreased at the protein level (Fig. 5D, *p* < 0.01) and mRNA level (Fig. 5E, *p* < 0.05). These partial experiments support that cisplatin may promote apoptosis of U2OS cells by downregulating PINK1, thus playing a role in killing osteosarcoma cancer cells.

## Discussion

There has been an increase of attention toward the function of PINK1 in the past decade following its discovery as an autosomal recessive gene involved in the pathogenesis of Parkinson’s disease [14]. Recently, PINK1 has been shown to affect cancer-related processes through mitophagy [15, 16]. At the same time, PINK1, as a cell cycle regulator, has the characteristics of promoting tumours and can significantly promote cell proliferation, colony formation, invasion and other cancer-related phenotypes [6, 17]. However, this gene has not been studied in osteosarcoma.

In this study, by analysing data from public databases, we found differential expression of PINK1 in a variety of cancers. More importantly, in osteosarcoma, the high expression of PINK1 suggests a worse prognosis. Research on PINK1 across cancers by Lizhe Zhu et al. [18] also confirmed its differential expression, as well as its different prognostic significance in different cancers. In addition, we analyzed the downstream pathways in which PINK1 may be involved, including apoptotic and proliferation pathways, as well as a series of coexpressed proteins, including FOXO3 and others. Previous studies have reported that FOXO3 can act as an upstream regulator of PINK1, thereby regulating mitophagy [19]. FOXO3 impaired PINK1 protein stability by driving BNIP3 transcriptional repression in hepatocellular carcinoma [20]. In COPD, SIRT1 activation can reduce FOXO3 acetylation, thereby increasing PINK1 protein levels and enhancing mitophagy [21]. In addition, FOXO3a regulates PINK1-dependent mitophagy and inflammasome activation through acetylation in diabetic macrophages [22]. By binding to the FOXO3a transcription factor, the DJ1 protein directly interacts with the PINK1 promoter and stimulates its transcriptional activity, thereby regulating cell metabolism and proliferation [23].

Based on the bioinformatics analysis and the results of other experiments, we demonstrate that PINK1 is upregulated in osteosarcoma at the protein and mRNA levels, suggesting that PINK1 plays an important role in the development of osteosarcoma. Previous studies have shown that PTEN induces PINK1 expression [24]. However, we found that PTEN expression is repressed in osteosarcoma, while PINK1 expression is promoted. Therefore, we further investigated the coexpression protein FOXO3a selected by bioinformatics and found that it was highly expressed in osteosarcoma. Previous research has shown that PINK1 is a downstream target of FOXO3a in cancers [25, 26]. A study on osteosarcoma also reported that the expression level of FOXO3a was significantly reduced, and the CtBP1-p300-FOXO3a transcription complex can inhibit the expression of apoptosis regulators in human osteosarcoma cells [27]. However, miR-29a-3p inhibits the progression of OS by inducing autophagy and inhibiting the IGF1-mediated FOXO3 pathway [28]. In the development of chemotherapeutic drugs, several drugs, such as mitoxantrone [29], acetylshikonin [30] and Brazil Lin [31], have been found to promote FOXO3-dependent cell death in osteosarcoma cells. Consistent with previous studies, our findings showed a positive correlation between FOXO3a and PINK1. FOXO3a, rather than PTEN, upregulates the expression of PINK1 in osteosarcoma.

PINK1 has cytoprotective effects by improving cancer cell proliferation and survival and prevents apoptosis in cancer cells [32]. Consistent with this study, we found that the knockdown of PINK1 in U2OS cells significantly suppresses cell proliferation and causes cell cycle arrest at G1/S. In addition, acridine orange (AO) can penetrate the intact cell membrane and embed into the nuclear DNA, causing it to emit bright green fluorescence. Ethidium bromide (EB) can penetrate the damaged cell membrane, embed into nuclear DNA, and emit orange fluorescence. In this study, we found that the nuclear chromatin of U2OS cells in the control group was green and showed a normal structure, while after PINK1 knockdown, the nuclear chromatin of U2OS cells was orange and showed a pyknotic or beadlike shape, suggesting that apoptosis was significantly enhanced. It has been reported that silencing PINK1 can effectively inhibit lung cancer and breast cancer cell proliferation and induce tumour cell apoptosis both in vivo and in vitro [33, 34]. Targeting PINK1-mediated mitophagy can improve the inflammatory response and apoptosis in myocardial ischaemia/reperfusion [35], inhibit apoptosis induced by astrosporin [36], and attenuate mitochondrial apoptosis induced by copper [37]. Pink1-deficient mice reprogram glucose metabolism by HIF1 to maintain cell proliferation [38]. High glucose promotes retinal pigment epithelial cell apoptosis and inhibits cell proliferation by regulating ROS-mediated inactivation of the ROS/PINK1/Parkin signalling pathway [39]. These results are all consistent with our results.

The treatment guidelines for advanced osteosarcoma [12, 40] are local radiotherapy and systemic chemotherapy after tumour resection. Currently, national and international cooperative trials for newly diagnosed OS patients are based on cisplatin, doxorubicin and methotrexate [41]. However, due to the side effects and high toxicity of cisplatin [13], bypassing cisplatin and directly acting on downstream target genes is the fundamental solution to avoid its toxicity.

Previous studies have confirmed that enhanced mitophagy mediated by high PINK1 expression can significantly enhance the sensitivity of hepatocellular carcinoma cells to radiotherapy [42] and lenvatinib [43]. In vitro studies have demonstrated that PINK1 promotes the proliferation of non-small cell lung cancer cells, and PINK1 expression is associated with stronger tumour invasion and poor prognosis. In addition, PINK1 knockdown has been found to enhance cisplatin-induced apoptosis in NSCLC cells [44]. Yancheng Tang et al. also suggest that targeting PINK1-mediated mitophagy may be a promising strategy to overcome chemotherapy resistance and improve anticancer therapy [45]. However, no study has reported the correlation between PINK1 and chemotherapy drugs in osteosarcoma. Our in vitro study showed that cisplatin can significantly promote the apoptosis of U2OS cells and downregulate the expression of PINK1 in OS cells, suggesting that cisplatin may regulate the apoptosis of OS cells through the PINK1 protein as a downstream target protein to achieve the effect of chemotherapy. However, our data did not clarify the exact action and mechanism of cisplatin on PINK1 in an animal model. In our study, only cisplatin was selected as a chemotherapy drug to treat OS cells, while other drugs, such as neomycin, were not administered. We will conduct in-depth studies on these two aspects in the future.

## Conclusions

Altogether, our results demonstrate that PINK1 is negatively regulated by FOXO3a and plays a regulatory role as a downstream target protein of cisplatin, promoting OS cell proliferation and inhibiting OS cell apoptosis. Our results reveal that PINK1 is a prospective novel target for osteosarcoma treatment.

## Data Availability

The datasets generated for this study can be found in the https://portal.gdc.cancer.gov.

## References

[1] Guillon MA, Mary PM, Brugière L, Marec-Bérard P, Pacquement HD, Schmitt C, Guinebretière JM, Tabone MD (2011). Clinical characteristics and prognosis of osteosarcoma in young children: a retrospective series of 15 cases. BMC Cancer.

[2] Lee RJ, Arshi A, Schwartz HC, Christensen RE (2015). Characteristics and prognostic factors of osteosarcoma of the jaws: a retrospective cohort study. JAMA Otolaryngol Head Neck Surg.

[3] Fan XL, Cai GP, Zhu LL, Ding GM (2015). Efficacy and safety of ifosfamide-based chemotherapy for osteosarcoma: a meta-analysis. Drug Des Dev Ther.

[4] Jones KB, Salah Z, Del Mare S, Galasso M, Gaudio E, Nuovo GJ, Lovat F, LeBlanc K, Palatini J, Randall RL (2012). miRNA signatures associate with pathogenesis and progression of osteosarcoma. Cancer Res.

[5] Zhang X, Wan G, Mlotshwa S, Vance V, Berger FG, Chen H, Lu X (2010). Oncogenic Wip1 phosphatase is inhibited by miR-16 in the DNA damage signaling pathway. Cancer Res.

[6] O'Flanagan CH, Morais VA, Wurst W, De Strooper B, O'Neill C (2015). The Parkinson's gene PINK1 regulates cell cycle progression and promotes cancer-associated phenotypes. Oncogene.

[7] Billia F, Hauck L, Konecny F, Rao V, Shen J, Mak TW (2011). PTEN-inducible kinase 1 (PINK1)/Park6 is indispensable for normal heart function. Proc Natl Acad Sci USA.

[8] Matheoud D, Sugiura A, Bellemare-Pelletier A, Laplante A, Rondeau C, Chemali M, Fazel A, Bergeron JJ, Trudeau LE, Burelle Y (2016). Parkinson's disease-related proteins PINK1 and parkin repress mitochondrial antigen presentation. Cell.

[9] Tang Z, Kang B, Li C, Chen T, Zhang Z (2019). GEPIA2: an enhanced web server for large-scale expression profiling and interactive analysis. Nucleic Acids Res.

[10] Sahin Y (2022). LncRNA H19 is a potential biomarker and correlated with immune infiltration in thyroid carcinoma. Clin Exp Med.

[11] Szklarczyk D, Gable AL, Lyon D, Junge A, Wyder S, Huerta-Cepas J, Simonovic M, Doncheva NT, Morris JH, Bork P (2019). STRING v11: protein–protein association networks with increased coverage, supporting functional discovery in genome-wide experimental datasets. Nucleic Acids Res.

[12] Smrke A, Anderson PM, Gulia A, Gennatas S, Huang PH, Jones RL (2021). Future directions in the treatment of osteosarcoma. Cells.

[13] Ghosh S (2019). Cisplatin: the first metal based anticancer drug. Bioorg Chem.

[14] McLelland GL, Soubannier V, Chen CX, McBride HM, Fon EA (2014). Parkin and PINK1 function in a vesicular trafficking pathway regulating mitochondrial quality control. Embo J.

[15] O'Flanagan CH, O'Neill C (2014). PINK1 signalling in cancer biology. Biochim Biophys Acta.

[16] Bernardini JP, Lazarou M, Dewson G (2017). Parkin and mitophagy in cancer. Oncogene.

[17] Dai K, Radin DP, Leonardi D (2021). Deciphering the dual role and prognostic potential of PINK1 across cancer types. Neural Regen Res.

[18] Zhu L, Wu W, Jiang S, Yu S, Yan Y, Wang K, He J, Ren Y, Wang B (2020). Pan-cancer analysis of the mitophagy-related protein PINK1 as a biomarker for the immunological and prognostic role. Front Oncol.

[19] Song D, Ma J, Chen L, Guo C, Zhang Y, Chen T, Zhang S, Zhu Z, Tian L, Niu P (2017). FOXO3 promoted mitophagy via nuclear retention induced by manganese chloride in SH-SY5Y cells. Metallomics Integr Biomet Sci.

[20] Yao J, Wang J, Xu Y, Guo Q, Sun Y, Liu J, Li S, Guo Y, Wei L (2022). CDK9 inhibition blocks the initiation of PINK1-PRKN-mediated mitophagy by regulating the SIRT1-FOXO3-BNIP3 axis and enhances the therapeutic effects involving mitochondrial dysfunction in hepatocellular carcinoma. Autophagy.

[21] Jiang H, Jiang Y, Xu Y, Yuan D, Li Y. Bronchial epithelial SIRT1 deficiency exacerbates cigarette smoke induced emphysema in mice through the FOXO3/PINK1 pathway. Experimental lung research, 1–16. Advance online publication; 2022. 10.1080/01902148.2022.2037169 35132913

[22] Gupta P, Sharma G, Lahiri A, Barthwal MK (2022). FOXO3a acetylation regulates PINK1, mitophagy, inflammasome activation in murine palmitate-conditioned and diabetic macrophages. J Leukoc Biol.

[23] Requejo-Aguilar R, Lopez-Fabuel I, Jimenez-Blasco D, Fernandez E, Almeida A, Bolaños JP (2015). DJ1 represses glycolysis and cell proliferation by transcriptionally up-regulating Pink1. Biochem J.

[24] Norris KL, Hao R, Chen LF, Lai CH, Kapur M, Shaughnessy PJ, Chou D, Yan J, Taylor JP, Engelender S (2015). Convergence of Parkin, PINK1, and α-synuclein on stress-induced mitochondrial morphological remodeling. J Biol Chem.

[25] Cardona F, Sánchez-Mut JV, Dopazo H, Pérez-Tur J (2011). Phylogenetic and in silico structural analysis of the Parkinson disease-related kinase PINK1. Hum Mutat.

[26] Devireddy S, Liu A, Lampe T, Hollenbeck PJ (2015). The Organization of mitochondrial quality control and life cycle in the nervous system in vivo in the absence of PINK1. J Neurosci.

[27] Li C, Xiao XQ, Qian YH, Zhou ZY (2019). The CtBP1-p300-FOXO3a transcriptional complex represses the expression of the apoptotic regulators Bax and Bim in human osteosarcoma cells. J Cell Physiol.

[28] Qi S, Xu L, Han Y, Chen H, Cheng A (2022). miR-29a-3p mitigates the development of osteosarcoma through modulating IGF1 mediated PI3k/Akt/FOXO3 pathway by activating autophagy. Cell Cycle.

[29] Park SH, Lee J, Kang MA, Jang KY, Kim JR (2018). Mitoxantrone induces apoptosis in osteosarcoma cells through regulation of the Akt/FOXO3 pathway. Oncol Lett.

[30] Cha HS, Lee HK, Park SH, Nam MJ (2023). Acetylshikonin induces apoptosis of human osteosarcoma U2OS cells by triggering ROS-dependent multiple signal pathways. Toxicol In Vitro.

[31] Kang Y, He P, Wang H, Ye Y, Li X, Xie P, Wu B (2018). Brazilin induces FOXO3A-dependent autophagic cell death by disturbing calcium homeostasis in osteosarcoma cells. Cancer Chemother Pharmacol.

[32] Kubli DA, Cortez MQ, Moyzis AG, Najor RH, Lee Y, Gustafsson ÅB (2015). PINK1 Is dispensable for mitochondrial recruitment of Parkin and activation of mitophagy in cardiac myocytes. PLoS ONE.

[33] Liu L, Zuo Z, Lu S, Wang L, Liu A, Liu X (2018). Silencing of PINK1 represses cell growth, migration and induces apoptosis of lung cancer cells. Biomed Pharmacother.

[34] Li J, Xu X, Huang H, Li L, Chen J, Ding Y, Ping J (2022). Pink1 promotes cell proliferation and affects glycolysis in breast cancer. Exp Biol Med.

[35] Yao L, Chen H, Wu Q, Xie K (2019). Hydrogen-rich saline alleviates inflammation and apoptosis in myocardial I/R injury via PINK-mediated autophagy. Int J Mol Med.

[36] Brunelli F, Torosantucci L, Gelmetti V, Franzone D, Grünewald A, Krüger R, Arena G, Valente EM (2022). PINK1 protects against staurosporine-induced apoptosis by interacting with Beclin1 and impairing its pro-apoptotic cleavage. Cells.

[37] Yang F, Liao J, Yu W, Qiao N, Guo J, Han Q, Li Y, Hu L, Pan J, Tang Z (2021). Exposure to copper induces mitochondria-mediated apoptosis by inhibiting mitophagy and the PINK1/parkin pathway in chicken (*Gallus gallus*) livers. J Hazard Mater.

[38] Requejo-Aguilar R, Lopez-Fabuel I, Fernandez E, Martins LM, Almeida A, Bolaños JP (2014). PINK1 deficiency sustains cell proliferation by reprogramming glucose metabolism through HIF1. Nat Commun.

[39] Zhang Y, Xi X, Mei Y, Zhao X, Zhou L, Ma M, Liu S, Zha X, Yang Y (2019). High-glucose induces retinal pigment epithelium mitochondrial pathways of apoptosis and inhibits mitophagy by regulating ROS/PINK1/Parkin signal pathway. Biomed Pharmacother.

[40] Liu X, He S, Wu H, Xie H, Zhang T, Deng Z (2019). Blocking the PD-1/PD-L1 axis enhanced cisplatin chemotherapy in osteosarcoma in vitro and in vivo. Environ Health Prev Med.

[41] Eastman A (2017). Improving anticancer drug development begins with cell culture: misinformation perpetrated by the misuse of cytotoxicity assays. Oncotarget.

[42] Yu L, Yang X, Li X, Qin L, Xu W, Cui H, Jia Z, He Q, Wang Z (2021). Pink1/PARK2/mROS-dependent mitophagy initiates the sensitization of cancer cells to radiation. Oxid Med Cell Longev.

[43] Zheng Y, Huang C, Lu L, Yu K, Zhao J, Chen M, Liu L, Sun Q, Lin Z, Zheng J, Chen J, Zhang J (2021). STOML2 potentiates metastasis of hepatocellular carcinoma by promoting PINK1-mediated mitophagy and regulates sensitivity to lenvatinib. J Hematol Oncol.

[44] Zhang R, Gu J, Chen J, Ni J, Hung J, Wang Z, Zhang X, Feng J, Ji L (2017). High expression of PINK1 promotes proliferation and chemoresistance of NSCLC. Oncol Rep.

[45] Tang Y, Wang L, Yi T, Xu J, Wang J, Qin JJ, Chen Q, Yip KM, Pan Y, Hong P, Lu Y, Shen HM, Chen HB (2021). Synergistic effects of autophagy/mitophagy inhibitors and magnolol promote apoptosis and antitumor efficacy. Acta pharmaceutica Sinica B.

